# Probabilistic Approach to Limit States of a Steel Dome

**DOI:** 10.3390/ma14195528

**Published:** 2021-09-24

**Authors:** Paweł Zabojszcza, Urszula Radoń, Waldemar Szaniec

**Affiliations:** Faculty of Civil Engineering and Architecture, Kielce University of Technology, 25-314 Kielce, Poland; zmbur@tu.kielce.pl (U.R.); waldek.szaniec@tu.kielce.pl (W.S.)

**Keywords:** steel dome, reliability analysis, ultimate limit states, serviceability limit state, reliability index, elasticity index

## Abstract

In this paper, Numpress Explore software, developed at the Institute of Fundamental Technological Research of the Polish Academy of Sciences (IPPT PAN), was used to conduct reliability analyses. For static-strength calculations, the MES3D module, designed by the authors, was employed. Ultimate limit state was defined as condition of non-exceedance of the capacity value, resulting from the stability criterion of the bent and compressed element. The serviceability limit state was defined as the condition of non-exceedance of allowable vertical displacement. The above conditions constitute implicit forms of random variable functions; therefore, it was necessary to build an interface between the Numpress Explore and MES3D programs. In the study, a comparative analysis of two cases was carried out. As regards the first case, all adopted random variables had a normal distribution. The second case involved a more accurate description of the quantities mentioned. A normal distribution can be adopted for the description of, e.g., the randomness in the location of the structure nodes, and also the randomness of the multiplier of permanent loads. In actual systems, the distribution of certain loads deviates substantially from the Gaussian distribution. Consequently, adopting the assumption that the loads have a normal distribution can lead to gross errors in the assessment of structural safety. The distribution of loads resulting from atmospheric conditions is decidedly non-Gaussian in character. The Gumbel distribution was used in this study to describe snow and wind loads. The modulus of elasticity and cross-sectional area were described by means of a log-normal distribution. The adopted random variables were independent. Additionally, based on an analysis of the elasticity index, the random variables most affect the failure probability in the ultimate limit state and serviceability limit state were estimated.

## 1. Introduction

In accordance with guidelines [1,2,3,4], a check of structural reliability is based on an idealized concept of limit states and their verification by means of a semi-probabilistic method of partial safety factors. A building structure is characterized by two excluding states, namely a fitness state, in which the object meets the set requirements, and an unfitness state, when it does not satisfy those requirements. The conditions that separate the fitness state from the unfitness state are termed limit states. The method mentioned above involves an analysis of the bearing capacity mobilization of members or sections (ULS—ultimate limit state) and of serviceability criteria (SLS—serviceability limit state). Uncertainties in materials parameters and loads are taken into account solely in the form of related, representative values that are called partial safety factors. Those factors are understood to be related quantiles of probability distributions that describe random variables. The role of partial safety factors is to ensure the required level of reliability of the structure. The fully probabilistic methods are extensions of semi-probabilistic method of limit states. These methods are divided on two groups: approximated and simulated methods. The first order reliability method (FORM) [5,6,7] and the second order reliability method (SORM) [8,9,10,11] are approximated approaches. Importance sampling [12,13,14], artificial intelligence [15,16], and the Monte Carlo method [17,18,19] are simulation approaches. A probabilistic approach makes it possible to provide a description that is more accurate and closer to the actual reality with respect to structure materials, geometric parameters, and loads. This is done by giving the type of probability distribution and distribution parameters.

In this paper, the first order reliability method was used to assess the limit states of the structure. The measure of structural reliability was the Hasofer-Lind reliability index [5]. The previously defined Cornell index [6] had a significant disadvantage—no invariance and it resulted from the position of the point for linearization as average values. This made the reliability measure difficult to use when comparing the safety of different structures. In 1978, the design point approximation algorithm was proposed by Rackwitz and Fiessler [7]. In [20], Hohenbichler and Rackwitz came up with an idea to apply the Rosenblatt transformation [21] to transform dependent random variables into a standard space. Der Kiureghian and Liu [22] employed the Nataf transformation to that end. The use of first- or second-order functions to approximate the limit state function at the design point leads to the first order reliability method (FORM) or the second order reliability method (SORM).

Early applications of reliability analysis methods assumed that the limit function was an explicit function of random variables. The presented dependence can only be implemented on simple examples. Unfortunately, in practical implementations, this dependence is implicit, which requires the use of a numerical procedure, for example, the finite element method (FEM). The development of numerical methods contributed to the use of very desirable tools in the theory of structure reliability, such as the methods of implicit dependence of the boundary state functions on basic random variables. An example is the perturbative method proposed in the works of Hisada and Nakagiri [23], Liu, Belytschko, and Mani [24], and Shinozuki [25] for tasks of the linear theory of elasticity. This method consists in expanding the stiffness matrix into a power series with respect to the random fluctuations of its parameters. However, the perturbative method is not intended to calculate the failure probability, but only to obtain statistical response moments. Calculating the probabilities would require introducing probability distributions of random variables into the calculations and estimating the higher moments of the statistical responses. Similar to the perturbative method, also using the Neumann expansion (the inverse matrix to the stiffness matrix is expanded in series), only information about the statistical moments of the random variable (being the response of the system) is obtained. Li and Der Kiureghian [26], and also Matthies [27] proposed a method that allows to estimate the probability of failure in a situation where the design is calculated using the finite element method.

In [28], Pelisetti and Schueller provided an overview of various software tools that enable rational treatment of the effects of uncertainty. The article covered issues that include the underlying algorithms, and also the interaction with deterministic FE codes. In addition, other topics were discussed, including the availability of graphical user interfaces, distributed processing, verification functions, and the possibility of probabilistic modeling. The work in [29] describes the problems that can be solved with two additional tools introduced by Ansys Inc.: ANSYS Probabilistic Design System and the ANSYS DesignXplorer. In both modules, an extremely efficient method can be used in which an accurate high-order response surface, using single finite element analysis, variation technology, is provided. Der Kiuregian et al. [30] discussed the Calrem/Ferum and OpenSees programs for the analysis of structural reliability, while the work in [31] presents an overview of the current state of COSSANe software. In [32], Thacker et al. discussed NESSUS probabilistic analysis software, which allows the user to combine advanced probabilistic algorithms with analytical equations, commercial finite element analysis programs, and their “own” standalone deterministic analysis codes to calculate probabilistic responses or system reliability. Gollwitzer et al. [33] described examples of analyses using a combination of the Strurel probabilistic analysis system, which includes Comrel, Sysrel, Costrel, and Statrel, and the PERMAS general purpose finite element program. The authors of [34,35] described the general-purpose probabilistic software PHIMECA and Proban. A summary of the possibilities of the program for probabilistic analysis and designing ProFES is presented in [36]. This program allows to perform a wide range of probabilistic function assessments, including finite element analysis in a graphical environment. UNIPASS is a probabilistic evaluation software that provides the ability to model uncertainty, define probability distribution variables, and probabilistic models and responses [37]. The use of reliability analysis and of the finite element method has been described by many researchers (Lee and Ang [38], Engelstad and Reddy [39], Mahadevan and Mehty [40], Zabojszcza and Radoń [41,42], Mochocki et al. [43], Kubicka et al. [44]).

When creating a program that enables reliability analysis through the use of the finite element method, the best situation is to have access to the FEM source code and a reliability program. In general, however, this is not possible; therefore, at the expense of efficiency loss, existing MES packages are combined with a reliability program, using various types of interfaces. The paper presents a combination of the Numpress reliability program with the MES3D proprietary module. There is resistance among engineers to use numerical probabilistic methods, the complexity of which is hidden inside computer programs. Additional effort required from the user of the program is the necessity to characterize data with two parameters, i.e., the expected value and the standard deviation, instead of the one parameter used in deterministic methods. For this reason, it is necessary to provide engineers with algorithms that will enable the analysis of a structure, taking into account random factors. According to the authors, the FORM method may fill this gap in the future.

## 2. Materials and Methods

### 2.1. Method FORM

**Figure 1:**
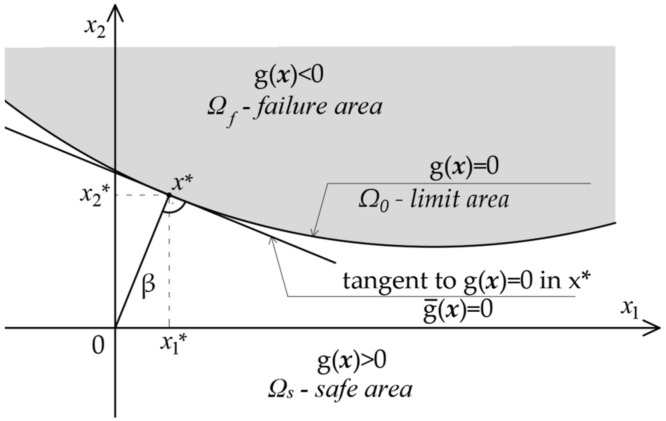
Non-linear, n-dimensional (for n = 2) surface of the limit state function g(**x**) = 0 realization space of the basic random variables, with the marking of linearization on the surface $\bar{g}\left(x\right)$ of the limit state function at point x*.

The increase in computational power allows to design more and more complex structures. Nowadays, static-strength analysis of structures is implemented by means of the finite element method (FEM). Nevertheless, a design procedure is still performed deterministically, applying safety factors. This concept aims to ensure that the risk induced due to the randomness of input parameters is sufficiently small. A more efficient way is take the impact of uncertainties in a structural response into account by performing structural reliability analyses. In reliability analysis, the probability of failure is calculated, accounting for randomness in the input parameters. Let the vector **X** denote a set of basic random variables. Let g(**X**) represents the design criterion (limit state function), with negative values defining the failure. Hasofer and Lind [5] presented the concept of the location of the so-called “design point”, i.e., such a realization of random variables (**x**) located in the failure area, which corresponds to the highest value of the probability density function. By linearizing the limit function at design point x*, a measure of reliability can be obtained which is invariable due to the equivalent formulation of the boundary condition, the so-called Hasofer–Lind reliability index, β. Figure 1 shows a graphical form of the problem of the reliability index determination for the non-linear limit state function g(**x**) = 0 in the realization space of basic random variables.

The transformation of the basic variables from the original to the standard normal space of uncorrelated random variables, *Z* = *T*(**X**), is used to facilitate the implementation of the algorithms. The precise determination of the design point is extremely important. There are many works aimed at comparing the effectiveness of different algorithms. Most of these come from researchers associated with the research laboratories of Rackwitz and Der Kiureghianna. Currently, due to their efficiency and ease of implementation, the most frequently used algorithms are based on the standard Rackwitz–Fiessler method. According to this algorithm, the task of locating a design point can be determined as follows:

find

(1) $$\min Q(z)=z^{T}z,$$

under the constraint

(2) g(z)=0.

After expanding the quadratic objective function (*Q*(***z***)) around point ***z***^(*k*)^ in a Taylor series, and also linearizing function *g*(***z***), we have the task of finding the optimal increment, ∆***z***^(*k*)^:

(3) $$\begin{array}{cc} \min\tilde{Q}\left(\Delta z^{\left(k\right)}\right) & =Q\left(z^{\left(k\right)}\right)+\nabla Q^{T}\left(z^{\left(k\right)}\right)\cdot \Delta z^{\left(k\right)}+\frac{1}{2}\Delta z^{(k)T}\cdot \nabla^{2}Q\left(z^{\left(k\right)}\right)\cdot \Delta z^{\left(k\right)}\\ & =z^{(k)T}z^{\left(k\right)}+2z^{(k)T}\Delta z^{\left(k\right)}+\Delta z^{(k)T}\Delta z^{\left(k\right)}, \end{array}$$

under the constraint

(4) $$\tilde{g}\left(\Delta z^{\left(k\right)}\right)=g\left(z^{\left(k\right)}\right)+\nabla g^{T}\left(z^{\left(k\right)}\right)\cdot \Delta z^{\left(k\right)}=0.$$

The condition of the existence of the local minimum at point ***z****^k^* results from the stationarity condition of the Lagrange function. The Lagrange function has the following form:

(5) $$L\left(\Delta z^{\left(k\right)},\lambda \right)=z^{(k)T}z^{\left(k\right)}+2z^{(k)T}\Delta z^{\left(k\right)}+\Delta z^{(k)T}\Delta z^{\left(k\right)}-\lambda \left(g\left(z^{\left(k\right)}\right)+\nabla g^{T}\left(z^{\left(k\right)}\right)\cdot \Delta z^{\left(k\right)}\right)\;.$$

The necessary condition is stated by the so-called Kuhn–Tucker criteria.

Kuhn–Tucker conditions are as follows:

(6) $$\nabla L=2z^{\left(k\right)}+2\Delta z^{\left(k\right)}-\lambda \nabla g\left(z^{\left(k\right)}\right)=0,$$

(7) $$g\left(z^{\left(k\right)}\right)+\nabla g^{T}\left(z^{\left(k\right)}\right)\cdot \Delta z^{\left(k\right)}=0.$$

After transformations, an iterative formula for finding a design point is obtained:

(8) $$z^{\left(k+1\right)}=\frac{1}{{\left\|\nabla g\left(z^{\left(k\right)}\right)\right\|}^{2}}\cdot \left(\nabla g^{T}\left(z^{\left(k\right)}\right)\cdot z^{\left(k\right)}-g\left(z^{\left(k\right)}\right)\right)\cdot \nabla g\left(z^{\left(k\right)}\right)\;.$$

Iterations are continued until the condition is met:

(9) $$\left|z_{i}^{\left(k+1\right)}-z_{i}^{\left(k\right)}\right|\leq \varepsilon \;\mathrm{for}\;\mathrm{all}\;i\;\mathrm{and}\;\left|g\left(z*\right)\right|\leq \varepsilon .$$

The relationship has a clear geometrical interpretation:

(10) $$z^{\left(k+1\right)}=\underset{\mathrm{length}}{\underbrace{\frac{1}{{\left\|\nabla g\left(z^{\left(k\right)}\right)\;\right\|}^{}}\cdot \left(\nabla g^{T}\left(z^{\left(k\right)}\right)\;z^{\left(k\right)}-g\left(z^{\left(k\right)}\right)\right)}}\;\underset{\begin{array}{l} \mathrm{search}\\ \mathrm{direction} \end{array}}{\underbrace{\frac{\nabla g\left(z^{\left(k\right)}\right)}{{\left\|\nabla g\left(z^{\left(k\right)}\right)\;\right\|}^{}}}}.$$

The length of vector ***z***^(*k*+1)^ is a sum of the length of the vector ***z***^(*k*)^ projection in the direction $\nabla g\left(z^{\left(k\right)}\right)$ and the length $g\left(z^{\left(k\right)}\right)/\left\|\nabla g\left(z^{\left(k\right)}\right)\;\right\|,$ which results from substituting the limit surface with a trace of hyperplane $g^{\prime }\left(z^{\left(k\right)}\right)=0$, the latter originating in the linearization of the limit function at point ***z***^(*k*)^.

Finding the design point is a key step in the FORM method. This is an approximation method, according to which the failure area is approximated by linearization of the limit state function at the design point. The distance of the most probable point of failure from the mean value of random variables is represented by reliability index β. On its basis, the probability of failure is approximated as:

P_f_ ≈ ϕ(−β) (11)

where ϕ(.) is the standard normal cumulative distribution function.

**Figure 2:**
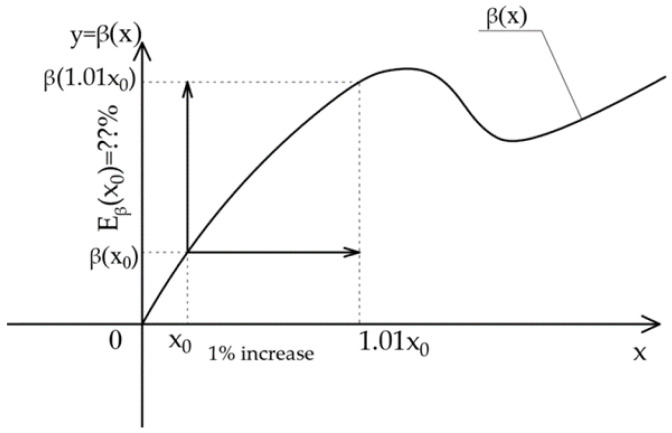
Illustration of the elasticity of the function β(x) of one variable [41].

A major advantage offered by the FORM method lies in the possibility of determining the reliability index sensitivity to the average values of random variables by calculating the elasticity index. It is practically not necessary to conduct any additional computations. The elasticity of the reliability index E_β_(x_0_) can be defined as a percentage change in reliability index β when parameter p_i_ is changed, e.g., by 1% (Stocki [45]) (Figure 2).

### 2.2. Crude Monte Carlo Method

The classical Monte Carlo method uses an independent random sample {X^(1)^, X^(2)^, …, X^(n)^}, obtained by generating random numbers directly from the probability distribution of a random variable modeling the parameters of the structure. Estimation of the failure probability, treated as the average value of the function:

(12) $${\bar{P}}_{\mathrm{fn}}=\frac{1}{n}\overset{n}{\underset{i=1}{\sum }}{I\Omega }_{f}\left(X^{\left(i\right)}\right),$$

which is called the empirical average value. Based on the strong law of large numbers, the sequence ${\bar{P}}_{\mathrm{fn}}$ converges with probability 1 to ${E(I\Omega }_{f}\left(X\right))$. The estimator error (12) is expressed by its variance, which in the case of the characteristic function of the failure area is defined by the expression:

(13) $$V_{P_{\mathrm{fn}}}=\mathrm{Var}\left({\bar{P}}_{\mathrm{fn}}\right)=\frac{1}{n}\;\int_{R^{n}}^{}{\left(I_{\Omega_{f}}\left(x\right)-E\left[I_{\Omega_{f}}\left(X\right)\right]\right)}^{2}f\left(x\right)\mathrm{dx}=\frac{1}{n}\left(E\left[I_{\Omega_{f}}{\left(X\right)}^{2}\right]-E{\left[I_{\Omega_{f}}\left(X\right)\right]}^{2}\right).$$

Estimation of $V_{P_{\mathrm{fn}}}$ on the basis of a random sample {X^(1)^, X^(2)^,…, X^(n)^} can be obtained using the estimator:

(14) $${\bar{V}}_{P_{\mathrm{fn}}}=\frac{1}{n^{2}}\overset{n}{\underset{i=1}{\sum }}{\left(I_{\Omega_{f}}{\left(X\right)}^{2}-{\bar{P}}_{\mathrm{fn}}\right)}^{2}.$$

The accuracy of the estimate (12) is often determined using the so-called the coefficient of variation of the estimator, defined as:

(15) $$\hat{e}=\frac{{\left({\bar{V}}_{P_{\mathrm{fn}}}\right)}^{\frac{1}{2}}}{{\bar{P}}_{\mathrm{fn}}}$$

An alternative to the determination of the point estimate and its error is to define the so-called the confidence interval in which the failure probability P_f_ is included with the assumed confidence level. The confidence interval of the estimator (12) can be determined using the fact that a random variable

(16) $$Y_{n}=\frac{\frac{1}{n}\sum_{i=1}^{n}{I\;\Omega }_{f}\left(X^{\left(i\right)}\right)-{E\;[I\;\Omega }_{f}\left(X\right)]}{\sqrt{V_{P_{\mathrm{fn}}}}},$$

by the central limit theorem, has an asymptotically standard normal distribution N(0,1). Let C be the assumed confidence level, and the k-quantile of the distribution N(0,1) of the order $\frac{1}{2}\left(1-C\right)$, then for n → ∞ there is $P\left(k\leq Y_{n}\leq -k\right)=C$.

Having the failure probability estimate (12) and its variance (15), based on Formulas (15) and (18), we obtain an approximation of the failure probability confidence interval:

(17) $$P\left({\bar{P}}_{\mathrm{fn}}+k\sqrt{\left({\bar{V}}_{P_{\mathrm{fn}}}\right)}\;\leq {\;P}_{f}\;\leq {\bar{\;P}}_{\mathrm{fn}}-k\sqrt{\left({\bar{V}}_{P_{\mathrm{fn}}}\right)}\right)=C.$$

The number of simulations that must be performed to obtain the estimator with the desired coefficient of variation can be determined from the formula:

(18) $$\hat{e}=\sqrt{\frac{1-{\bar{P}}_{\mathrm{fn}}}{{n\bar{P}}_{\mathrm{fn}}}},$$

Which you get by substituting expression (14) in (16); as can be seen, the number of necessary simulations does not depend on the number of dimensions of the random variable, which is one of the advantages of the classic Monte Carlo method. However, the error of the method is inversely proportional to the size of the probability to be estimated. To achieve the variability level of the estimate $\hat{e}=0.1$ for $P_{f}=3.16\times 10^{-5}$, which corresponds to the reliability index β = 4, more than $3\times 10^{6}$ simulations should be performed. Especially when the calculation of the failure function is associated with the performance of the finite element analysis, the numerical costs of the classic Monte Carlo method are very high. Despite this, the classic Monte Carlo formulation finds practical application due to the simplicity of its implementation.

### 2.3. Cooperation between Numpress Explore and MES3D Software

In this paper, Numpress Explore software developed at the Institute of Fundamental Technological Research of the Polish Academy of Sciences (IPPT PAN) [46] was used to conduct the reliability analysis. The website from which is it possible to download the software can be found in [47]. Solving reliability analysis issues requires the development of software that enables easy communication with external MES modules. Numpress’s object-oriented architecture software meets this requirement. The Numpress code is a collection of C++ libraries that group data classes and algorithms together. In addition, the code is equipped with mechanisms for interactive task definition. The procedure begins by creating a model of a stochastic task. The program user specifies the parameters of the boundary probability distributions of basic random variables. Then, the external variables are defined. External variables are implicit functions of the basic random variables. The values of external variables are obtained as a result of the FEM calculation modules for successive implementations of the vector of basic random variables. After defining the stochastic model via the graphical interface, the user enters the formula for the limit function. The limit function can contain both basic random variables and external variables. The next step is to choose the reliability analysis method and run the calculations. The task ends by generating a report containing the failure probability values and its sensitivity to the parameters of the probability distributions of random variables.

For static-strength calculations, the MES3D module, designed by the authors, was employed. The MES3D program was developed at the Kielce University of Technology [44]. It is constantly being developed in terms of computational capabilities. It was written in Object Pascal in the Lazarus IDE, which is based on the classical finite element method. It enables obtaining static solutions, natural vibrations, performing dynamic calculations—integration of equations of motion and simulations of fire problems. By default, in the standard mode, it is an interactive program. This especially applies to the visualization of the obtained solutions. However, in the article uses the ability to work in the console mode: the names of files containing data and the results of calculations were the parameters of the program. These are text files with a strictly defined structure, thanks to which it is possible to indicate the exact location of selected parameters used in the reliability analysis. This applies to both data and results. In the case of work in the console mode, graphic libraries were not loaded, which significantly accelerated the performance of proper numerical calculations.

**Figure 3:**
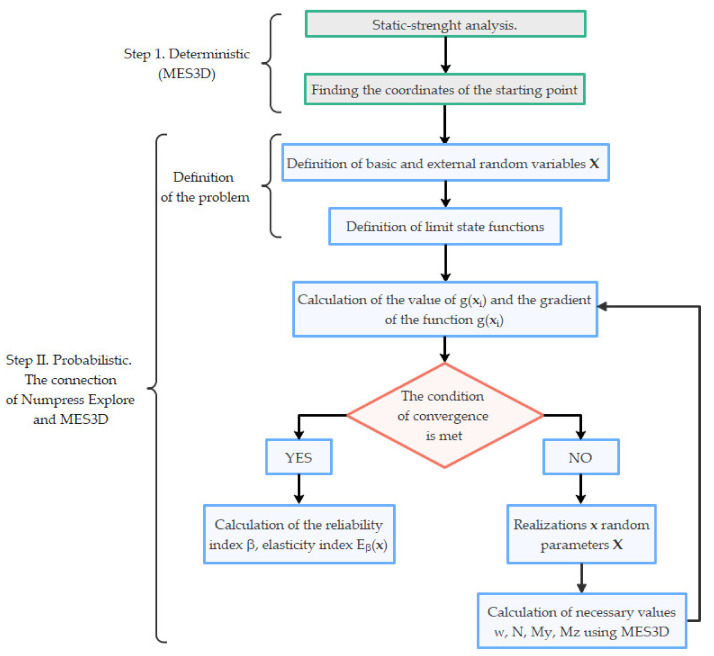
Cooperation between the Numpress Explore and MES3D software.

In this paper, external variables are vertical displacements, a bending moment with respect to the z-z axis, a bending moment with respect to the y-y axis, and axial forces. The basic random variables are dead load, snow load, wind load, Young’s modulus, and cross-sectional area. The ultimate limit state was defined as the condition of non-exceedance of the capacity value, resulting from the stability criterion of the bent and compressed element. The serviceability limit state was defined as the condition of non-exceedance of allowable vertical displacement. The above conditions constitute implicit forms of random variable functions; therefore, it was necessary to build an interface between the Numpress Explore and MES3D programs. Numpress Explore software communicates and controls the deterministic analyses by running the corresponding MES3D modules. The communication of the Numpress Explore program with external computing modules is ensured via text files. The realizations of random variables are saved in text input files. The MES3D program calculates the values necessary to define the limit state function for individual realizations of random variables. The calculation results of the MES3D program are read from the appropriate text output files. Figure 3 shows the algorithm for cooperation between Numpress Explore and MES3D software.

## 3. Results

### 3.1. Deterministic Static and Strength Analysis

**Figure 4:**
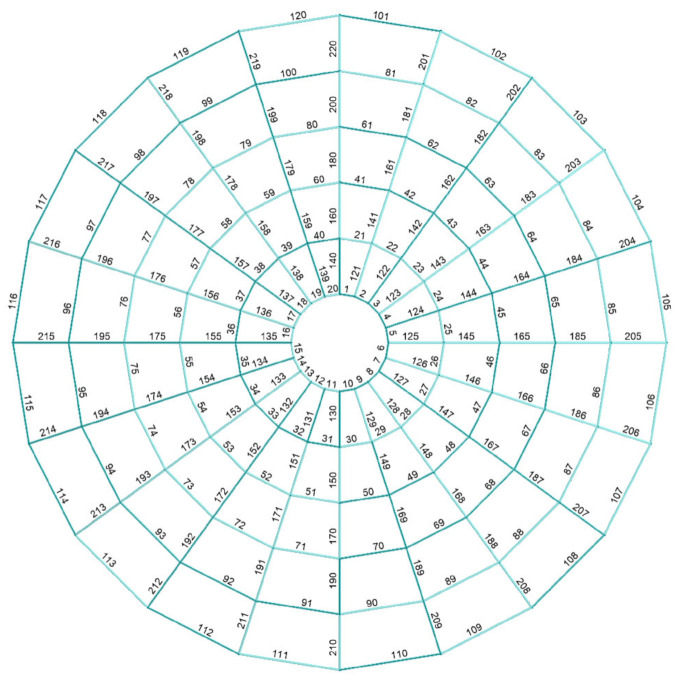
Ribbed dome.

The geometry of a steel lattice dome, consisting of 120 nodes and 220 elements, is presented in Figure 4 and in Table 1. The bars of the structure are modeled as frame elements. It was assumed that the structure elements are made of RO159x11 steel pipes with a yield point of fy = 235 MPa and Young’s modulus E = 210 GPa. The structure of the dome is based on 20 reinforced concrete columns, 5 m high, at the ends of which there is a reinforced concrete wreath that closes and stiffens the structure, and simulating the work of the grate.

**Figure 5:**
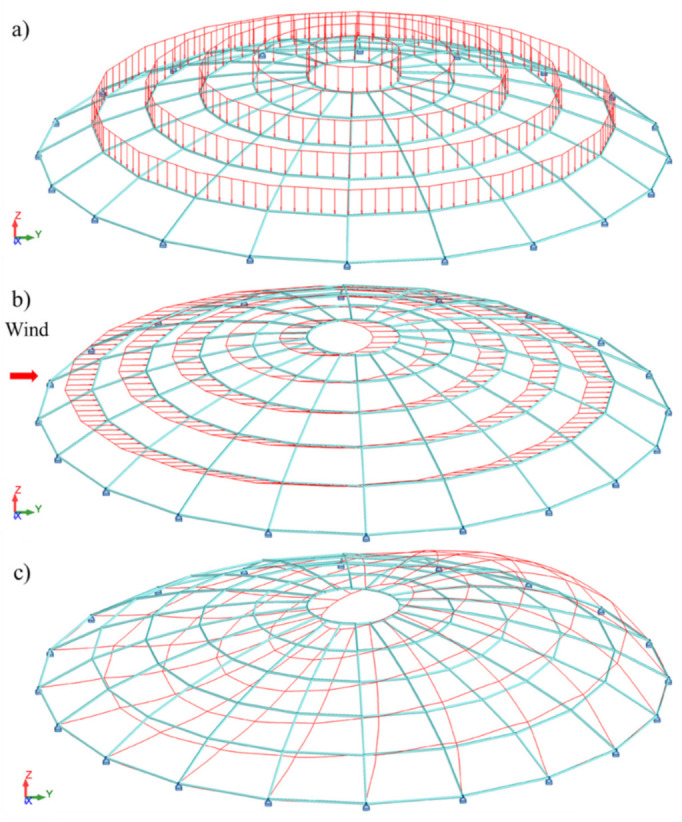
(**a**) Permanent load and snow load, (**b**) wind load, and (**c**) ribbed dome deformation.

The considered dome was loaded with a permanent load (self-weight of the bar structure and covering), a snow load determined for zone 3 according to [1], and a wind load according to [2] (Figure 5). The combination of loads applied to the structure was consistent with [3]. The deformation of the structure is shown in Figure 5c.

In the MES3D program, self-weight can be added automatically as a load evenly distributed on the bar or planar elements. In the calculations, it is also possible to take into account the additional weight of the covering elements, fittings, etc. In a similar way, the snow load is taken into account—in this case, it was collected from adjacent areas and added to the load on latitudinal elements.

**Figure 6:**
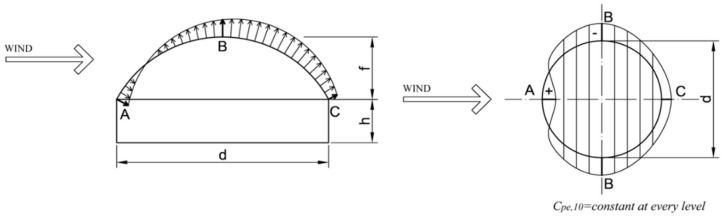
Standard wind pressure distribution of a circular dome [2].

In the case of circular domes, for wind loads, the PN-EN 1991-1-4 standard recommends assuming surface loads acting in the direction normal to the surface of the sphere. These values are constant in each arc resulting from the intersection of the canopy with the surface perpendicular to the wind direction (Figure 6 [2]).

However, even in the case of simple circular domes, the above definition causes a lot of problems in modeling; therefore, in practical implementations, various simplifying procedures are used, e.g., the surface is divided into zones, assuming constant load values in them.

**Figure 7:**
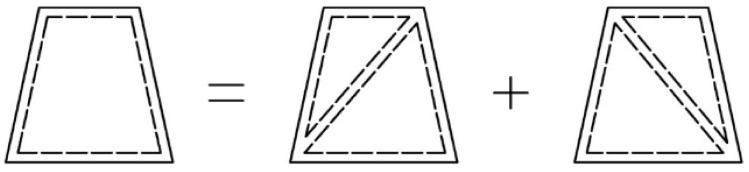
Diagram of applying wind load on 4-node elements.

For the dome under consideration, based on the geometry and geographic location, the values of the pressure coefficients at points A, B and C were determined. Values in the remaining points in the direction of the wind axis were determined using parabolic approximation. It was assumed that the load was applied to flat surface elements formed by the dome bars (Figure 4) and inside them it had a constant value defined for the element’s center of gravity. Triangular shell elements were used to transfer the load from the element surface to the nodes. For four-node areas, the load was divided into two equal parts, and then transferred to mesh nodes through triangular elements with different meshing (Figure 7). This modeling method does not introduce additional errors related to the use of triangular elements.

A static-strength analysis, on the basis of which the structure was dimensioned, was made according to [4] using MES3D software with spatial frameworks. In order to improve the accuracy of calculations, all elements were further divided into 10 parts.

**Figure 8:**
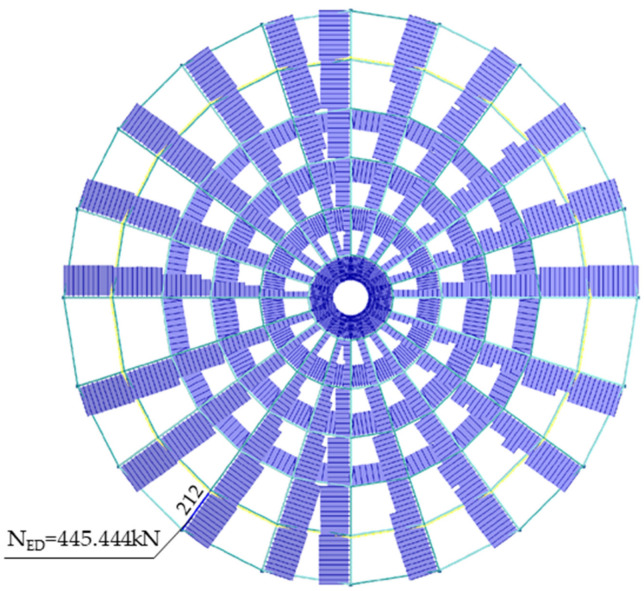
Axial force, N_Ed_.

**Figure 9:**
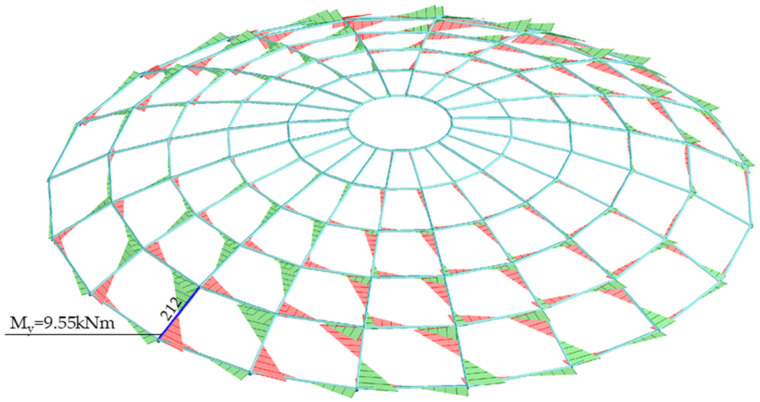
Bending moment with respect to the y-y axis My.

**Figure 10:**
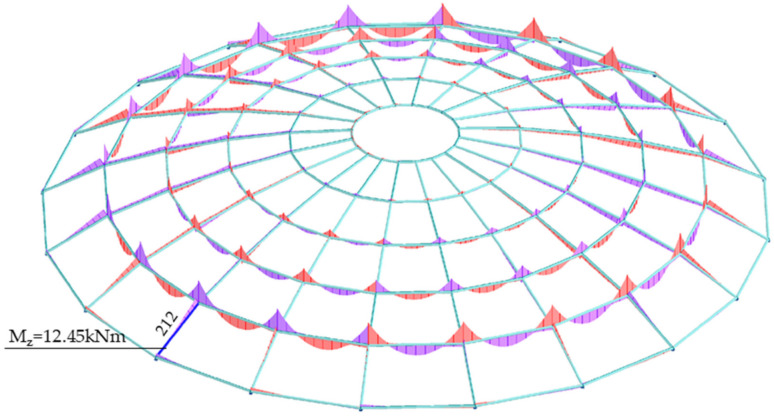
Bending moment with respect to the z-z axis Mz.

Internal forces and strength utilization of individual bars of the structure were specified (Figure 8, Figure 9 and Figure 10). Based on the static-strength analysis, it was observed that the most stressed bar was bar number 212. The maximum vertical displacement concerns node 58. The values of the internal forces, the capacity for the most stressed element of the structure (bar no. 212 in the meridian line), and the maximum horizontal and vertical displacement for node 58 are collated in Table 2. In the dimensioning of the structure, the case that turned out to be decisive was the one in which the permanent load, governing snow load and accompanying wind load were combined. As regards the selection of cross-sections, it was decided by the stability condition of the bent and compressed element.

The limit values in Table 2 have been determined in accordance with the standard PN-EN 1993-1-1. Eurocode 3: Design of steel structures. Part 1-1: General rules and rules for buildings. Figure 8 shows the distribution of axial forces with the distinction of the bar 212, for which the value is 445.444 kN.

The value of the My bending moment with respect to the y-y axis for bar no. 212 was: My = 9.55 kNm. Figure 9 shows diagrams of the bending moment with respect to the y-y axis.

The value of the Mz bending moment with respect to the z-z axis for bar no. 212 was: Mz = 12.45 kNm. Figure 10 shows diagrams of the bending moment with respect to the z-z axis.

### 3.2. Computation of the Hasofer-Lind Reliability Index

The next stage involved the analysis of the limit states of the single layer ribbed dome, in which a full probabilistic description was utilized. In the literature, many studies can be found in which the deterministic approach was adopted to describe the ultimate and serviceability limit states [48,49,50,51,52,53,54]. According to the deterministic approach, loads, material, and geometry features of the structure are described with code-specified characteristic values and relevant safety factors. The probabilistic approach offers a description of the above-mentioned quantities that is more accurate and closer to reality. It is possible to achieve that by providing the type and parameters of distribution.

The most frequently applied distribution in the probability theory is the normal distribution. It can be adopted to describe, e.g., the randomness of the location of structure nodes or of a permanent load multiplier. In actual systems, distributions of some loads significantly diverge from the Gaussian distribution. As a result, the adoption of the assumption of their being normal may lead to gross errors in the assessment of structure reliability. Weather-related loads are highly non-Gaussian in character. The Gumbel distribution was used to describe snow and wind loads. The modulus of elasticity and cross-sectional area were described by means of a log-normal distribution. In the study, a comparative analysis of two cases was conducted. For Case 1, all adopted random variables had a normal distribution. Case 2 accounted for a more accurate description of the quantities of concern. The adopted random variables were independent. Their descriptions are shown in Table 3 and Table 4.

The normal distribution is characterized by the following density function:

(19) $$f\left(x\right)=\frac{1}{\sigma \sqrt{2\pi }}\exp\left[-\frac{1}{2}{\left(\frac{x-\mu }{\sigma }\right)}^{2}\right];\;x\in \left(-\infty ,\infty \right)$$

Function (19) is defined by two parameters, namely by mean value:

(20) $$\mu =\overset{\infty }{\underset{-\infty }{\int }}x\;f\left(x\right)\mathrm{dx}$$

and by variance:

(21) $$\sigma^{2}=\overset{\infty }{\underset{-\infty }{\int }}{\left(x-\mu \right)}^{2}\;f\left(x\right)\mathrm{dx}$$

The Gumbel distribution is characterized by the following density function:

(22) $$f\left(x\right)=\alpha \;\exp\left[-\alpha (x-u)-\exp\left(-\alpha (x-u)\right)\right];\;x\in \left(-\infty ,\infty \right)$$

where α, u are parameters of the Gumbel distribution. The mean value and standard deviation are equal to μ=u+0.5772/α and σ=π/2.4495 α.

The lognormal distribution is characterized by the following density function:

(23) $$f\left(x\right)=\frac{1}{x\;\delta \;\sqrt{2\pi }}\exp\left[-\frac{1}{2}{\left(\frac{\ln(x/\xi )}{\delta }\right)}^{2}\right];\;x\in \left(0,\infty \right)$$

where $\delta^{2}=\ln\left({\mathrm{Cov}}^{2}+1\right),\;\xi =\frac{\bar{x}}{\sqrt{{\mathrm{Cov}}^{2}+1}},\;\mathrm{Cov}=\frac{\sigma \;}{\bar{x}}$.

In this work, it necessary to transform the normal distribution into a Gumbel or log-normal distribution; to achieve that, Method of Moments was used [55].

As regards the two-parameter distributions utilized in the paper, the method of probabilistic moments is based on the use of conformity conditions of the mean value and the variance. For the two-parameter distributions A and B, from the conformity conditions of the mean value and the variance, the following equations were developed:

(24) $$\left\{\begin{array}{c} \overset{\infty }{\underset{-\infty }{\int }}{\mathrm{xf}}_{A}\left(x\right)\mathrm{dx}=\overset{\infty }{\underset{-\infty }{\int }}{\mathrm{xf}}_{B}\left(x\right)\mathrm{dx}\\ \overset{\infty }{\underset{-\infty }{\int }}{\left({x-\mu }_{A}\right)}^{2}f_{A}\left(x\right)\mathrm{dx}=\overset{\infty }{\underset{-\infty }{\int }}{\left({x-\mu }_{B}\right)}^{2}f_{B}\left(x\right)\mathrm{dx} \end{array}\right.$$

Serviceability limit state SLS was considered as the condition of non-exceedance of the allowable vertical displacement:

g_SLS_ = 1 − abs(w)/(D/300), (25)

where w—external variable describing the maximum displacement of the structure, D—dome diameter.

Ultimate limit state (ULS) was defined as condition of non-exceedance of the capacity value, resulting from the stability criterion of the bent and compressed element:

g_ULS_ = 1 − N/N_b,Rd_ − k_zy_M_y_/χM_y,Rk_ − k_zz_M_z_/M_z,Rk_, (26)

where N [kN]—axial force, My [kNm]—bending moment with respect to the y-y axis, Mz [kNm]—bending moment with respect to the z-z axis, N_b,Rd_ [kN]—buckling resistance of the compressed element, M_y,Rk_ [kNm]—bending resistance with respect to the y-y axis, M_z,Rk_ [kNm]—bending resistance with respect to the z-z axis, χ—buckling coefficient, k_zy_, k_zz_—interaction coefficient according to [4].

The values of w, N, M_y_ and M_z_ are external variables that are computed by the MES3D software. For the applied functions of ULS and SLS, the Hasofer–Lind reliability index was computed using the FORM method. The FORM method was employed as a primary research tool. In order to validate the correctness of computation the Monte Carlo method was used (Table 5 and Table 6). The obtained values of the probability of failure are within the range 10^−3^–10^−5^. The necessary number of simulations was 10^9^. Due to the large number of simulations needed, only SLS was verified.

### 3.3. Analysis of the Sensitivity of the Hasofer-Lind Index to Changes in Random Parameters

**Figure 11:**
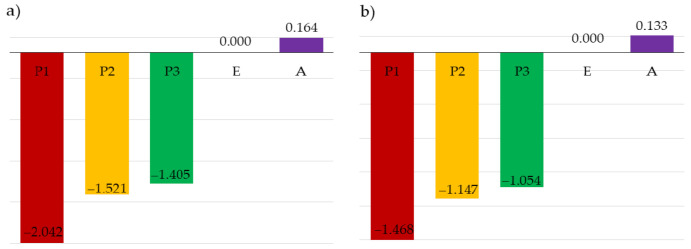
Elasticity index for ULS: (**a**) Case 1, (**b**) Case 2.

**Figure 12:**
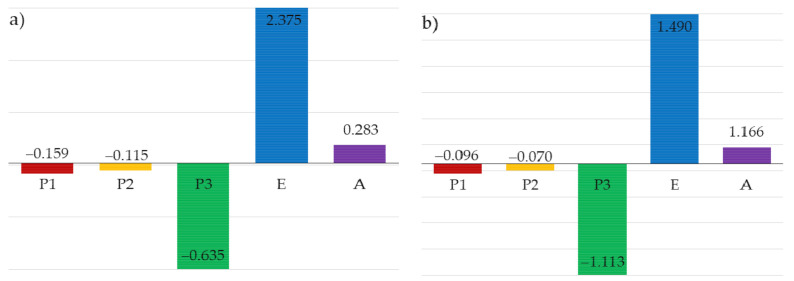
Elasticity index for SLS: (**a**) Case 1, (**b**) Case 2.

Additionally, based on the analysis of the elasticity index, which random variables most affect the failure probability in the ultimate limit state and serviceability limit state was estimated. When constructing the mathematical model of the task, the design engineer has to make a decision of which design parameters should be treated as random ones, and which as deterministic ones. With respect to ULS, the analysis of the elasticity index made it possible to estimate that random variables describing the permanent load, wind load, and snow load had the highest impact on the assessment of the structure safety (Figure 11). When SLS was considered, wind load and Young’s modulus were of the greatest relevance (Figure 12).

### 3.4. Discussion of the Results

In the study, two cases were examined that differed with respect to the type of probability distribution of the adopted random variables. In both cases, the possibility of stability failure was accounted for. Instability resulted from the exceedance of the allowable displacement (SLS) and the exceedance of the capacity condition of bent and compressed bar (ULS). Substantial differences in the value of ] reliability index β were observed, depending on the case considered and the limit state. The maximum value of the reliability index, namely 4.334, was obtained for SLS, when all the random variables of concern were accounted for and normal probability distribution was assumed. For Case 2, even when the limit state was the same, this value was reduced by 17.21%. In both cases, for ULS, the values of the reliability index were lower than those obtained for SLS. For Case 1 and Case 2, the index values amounted to 2.738 and 2.492, respectively. Based on these results, it can be observed that the identification of variables decides, to a great extent, the solution to the problem. For that reason, it is essential to examine the sensitivity of the reliability index to changes in the probabilistic characteristics of the random variables under consideration. That is done by means of specifying the elasticity index. Substantial differences in the determined values of the elasticity index were observed for the cases of concern. However, the same variables are decisive in both limit states for both cases. As regards ULS, the decisive effect of loads (random variables P1, P2, P3), especially of the dead load (variable P1), on the values of the reliability index can be seen. For SLS, however, the decisive variables are Young’s modulus—E and wind load (random variable P3). The results obtained with the Monte Carlo method agreed with the results obtained with the FORM method.

## 4. Conclusions

Two global parameters determine the safety of a structure: load and bearing capacity. Each of these parameters are random. The likelihood of failure is an objective, probabilistic measure of the safety of a structure. However, this measure is still not accepted by engineers. They prefer a security measure with a deterministic overtone, which is adopted in the semi-probabilistic method of limit states. It assesses the safety of a structure on the basis of the quantiles of the values of the characteristic loads and bearing capacity, as well as partial safety factors. The safety factors were separately calibrated for loads and bearing capacities. The random nature of load variability was taken into account by increasing them with an appropriate factor. The bearing capacity was assessed by its reduction. In the design of the limit state method, all possible design situations should be considered and it must be shown that none of the limit states was exceeded. This structural safety assessment is qualitative. The fully probabilistic methods are extensions of semi-probabilistic method of limit states. These methods allow the quantitative assessment of structure reliability.

Therefore, it is necessary to provide engineers with algorithms that enable the analysis of structures, taking into account random factors, which is not an easy task. Commonly available programs for static strength analysis using the finite element method are based on the deterministic limit state method. It is a good idea to try to combine existing design software with reliability analysis programs. The authors presented this path by combining MES3D with Numpress and showing other solutions of this type that are available.

In reliability analyses, it is crucial to adopt a computational model that represents real conditions as closely as possible. Incomplete statistical data or inappropriate assumptions may lead to substantial differences in the reliability index values. The study indicate that for the reliability analysis it is important to appropriately select not only random variables but also the type of their probability distribution.

It is highly advisable to conduct the analysis of the sensitivity of the assumed random variables. A important moment in the design process is to decide which parameter to treat as deterministic and which as random. The FORM method gives a quick answer to this question by analyzing the sensitivity of the reliability index.

## Figures and Tables

**Table 1:** Dome geometry.

No.	X	Y	Z	No.	X	Y	Z	No.	X	Y	Z
1	−3.380	0.000	6.000	41	−8.996	−6.536	4.700	81	−5.828	−17.937	2.200
2	−3.215	1.044	6.000	42	−10.576	−3.436	4.700	82	−11.086	−15.258	2.200
3	−2.734	1.987	6.000	43	−11.120	0.000	4.700	83	−15.258	−11.086	2.200
4	−1.987	2.734	6.000	44	−10.576	3.436	4.700	84	−17.937	−5.828	2.200
5	−1.044	3.215	6.000	45	−8.996	6.536	4.700	85	−18.860	0.000	2.200
6	0.000	3.380	6.000	46	−6.536	8.996	4.700	86	−17.937	5.828	2.200
7	1.044	3.215	6.000	47	−3.436	10.576	4.700	87	−15.258	11.086	2.200
8	1.987	2.734	6.000	48	0.000	11.120	4.700	88	−11.086	15.258	2.200
9	2.734	1.987	6.000	49	3.436	10.576	4.700	89	−5.828	17.937	2.200
10	3.215	1.044	6.000	50	6.536	8.996	4.700	90	0.000	18.860	2.200
11	3.380	0.000	6.000	51	8.996	6.536	4.700	91	5.828	17.937	2.200
12	3.215	−1.044	6.000	52	10.576	3.436	4.700	92	11.086	15.258	2.200
13	2.734	−1.987	6.000	53	11.120	0.000	4.700	93	15.258	11.086	2.200
14	1.987	−2.734	6.000	54	10.576	−3.436	4.700	94	17.937	5.828	2.200
15	1.044	−3.215	6.000	55	8.996	−6.536	4.700	95	18.860	0.000	2.200
16	0.000	−3.380	6.000	56	6.536	−8.996	4.700	96	17.937	−5.828	2.200
17	−1.044	−3.215	6.000	57	3.436	−10.576	4.700	97	15.258	−11.086	2.200
18	−1.987	−2.734	6.000	58	0.000	−11.120	4.700	98	11.086	−15.258	2.200
19	−2.734	−1.987	6.000	59	−3.436	−10.576	4.700	99	5.828	−17.937	2.200
20	−3.215	−1.044	6.000	60	−6.536	−8.996	4.700	100	0.000	−18.860	2.200
21	−6.895	−2.240	5.500	61	−8.811	−12.127	3.600	101	0.000	−22.730	0.000
22	−7.250	0.000	5.500	62	−12.127	−8.811	3.600	102	−7.024	−21.618	0.000
23	−6.895	2.240	5.500	63	−14.256	−4.632	3.600	103	−13.360	−18.389	0.000
24	−5.865	4.261	5.500	64	−14.990	0.000	3.600	104	−18.389	−13.360	0.000
25	−4.261	5.865	5.500	65	−14.256	4.632	3.600	105	−21.618	−7.024	0.000
26	−2.240	6.895	5.500	66	−12.127	8.811	3.600	106	−22.730	0.000	0.000
27	0.000	7.250	5.500	67	−8.811	12.127	3.600	107	−21.618	7.024	0.000
28	2.240	6.895	5.500	68	−4.632	14.256	3.600	108	−18.389	13.360	0.000
29	4.261	5.865	5.500	69	0.000	14.990	3.600	109	−13.360	18.389	0.000
30	5.865	4.261	5.500	70	4.632	14.256	3.600	110	−7.024	21.618	0.000
31	6.895	2.240	5.500	71	8.811	12.127	3.600	111	0.000	22.730	0.000
32	7.250	0.000	5.500	72	12.127	8.811	3.600	112	7.024	21.618	0.000
33	6.895	−2.240	5.500	73	14.256	4.632	3.600	113	13.360	18.389	0.000
34	5.865	−4.261	5.500	74	14.990	0.000	3.600	114	18.389	13.360	0.000
35	4.261	−5.865	5.500	75	14.256	−4.632	3.600	115	21.618	7.024	0.000
36	2.240	−6.895	5.500	76	12.127	−8.811	3.600	116	22.730	0.000	0.000
37	0.000	−7.250	5.500	77	8.811	−12.127	3.600	117	21.618	−7.024	0.000
38	−2.240	−6.895	5.500	78	4.632	−14.256	3.600	118	18.389	−13.360	0.000
39	−4.261	−5.865	5.500	79	0.000	−14.990	3.600	119	13.360	−18.389	0.000
40	−5.865	−4.261	5.500	80	−4.632	−14.256	3.600	120	7.024	−21.618	0.000

**Table 2:** Values of the internal forces, capacity of the most stressed element of the structure (bar no. 212 in the meridian line), and the maximum horizontal and vertical displacement of node 58.

Force/Bearing Capacity	Value
N_Ed_ [kN]—axial force	445.444
N_c,Rd_ [kN]—design capacity of the section under uniform compression	1200.85
N_b,Rd_ [kN]—design buckling resistance of the compressed element	878.945
M_y,Ed,Max_ [kNm]—design bending moment with respect to y-y axis	9.55
M_y,c,Rd_ [kNm]—design bending resistance with respect to y-y axis	56.73
M_z,Ed,max_ [kNm]—design bending moment with respect to z-z axis	12.45
M_z,c,Rd_ [kNm]—design bending resistance with respect to z-z axis	56.73
Strength utilization [%]	95
Maximum vertical displacement [mm]—for node 58	121.36
Allowable vertical displacement [mm]—D/300	151.53
Maximum horizontal displacement [mm]—for node 58	35.24
Allowable horizontal displacement [mm]—H/150	40.00

**Table 3:** Description of random variables for Case 1.

	Case 1
Random variable	Type of probability distribution
P1—dead load	Normal (5400, 540)
P2—snow load	Normal (3900, 562)
P3—wind load	Normal (300, 60)
E—Young’s modulus	Normal (2.1 × 10^11^, 5.1 × 10^9^)
A—cross-sectional area	Normal (0.00511, 0.0002555)

**Table 4:** Description of random variables for Case 2.

	Case 2
Random variable	Type of probability distribution
P1—dead load	Normal (5400, 540)
P2—snow load	Gumbel (3647.07, 438.190)
P3—wind load	Gumbel (272.997, 46.782)
E—Young’s modulus	Log-normal (2.1 × 10^11^, 5.1 × 10^9^)
A—cross-sectional area	Log-normal (0.00511, 0.0002555)

**Table 5:** Reliability index β for SLS.

	Reliability Index β for SLS	
	FORM	Monte Carlo
CASE 1	4.334	4.265
CASE 2	3.588	3.494

**Table 6:** Reliability index β for ULS.

	Reliability Index β for ULS
	FORM
CASE 1	2.738
CASE 2	2.492

## Data Availability

Not applicable.

## References

[1] EU (2003). PN-EN 1991-1-3: Eurocode 1: Actions on Structures—Part 1-3: General Actions—Snow Loads.

[2] EU (2010). PN-EN 1991-1-4: Eurocode 1: Actions on Structures—Part 1-4: General Actions—Wind Actions.

[3] EU (2005). PN-EN 1990:2004. Eurocode: Basis of Structural Design.

[4] EU (2005). PN-EN 1993-1-1. Eurocode 3: Design of Steel Structures. Part 1-1: General Rules and Rules for Buildings.

[5] Hasofer A.M., Lind N.C. (1974). Exact and Invariant Second-Moment Code Format. J. Eng. Mech. Div..

[6] Cornell C.A. (1969). A probability-based structural code. J. Am. Concr. Inst..

[7] Rackwitz R., Fiessler B. (1978). Structural reliability under combined random load sequences. Comput. Struct..

[8] Hohenbichler M., Gollwitzer S., Kruse W., Rackwitz R. (1987). New light on first- and second-order reliability methods. Struct. Saf..

[9] Fiessler B., Neumann H.-J., Rackwitz R. (1979). Quadratic Limit States in Structural Reliability. J. Eng. Mech. Div..

[10] Der Kiureghian A., De Stefano M. (1991). Efficient Algorithm for Second-Order Reliability Analysis. J. Eng. Mech..

[11] Der Kiureghian A., Lin H., Hwang S. (1987). Second-Order Reliability Approximations. J. Eng. Mech..

[12] Fujita M., Rackwitz R. (1988). Updating first-and second-order reliability estimates by importance sampling. Doboku Gakkai Ronbunshu.

[13] Doliński K. (1988). Importance Sampling Techniques in Reliability Calculations.

[14] Hohenbichler M., Rackwitz R. (1988). Improvement of Second-Order Reliability Estimates by Importance Sampling. J. Eng. Mech..

[15] Le L.M., Ly H.-B., Pham B.T., Le V.M., Pham T.A., Nguyen D.-H., Tran X.-T., Le T.-T. (2019). Hybrid Artificial Intelligence Approaches for Predicting Buckling Damage of Steel Columns Under Axial Compression. Materials.

[16] Dudzik A., Potrzeszcz-Sut B. (2021). Hybrid Approach to the First Order Reliability Method in the Reliability Analysis of a Spatial Structure. Appl. Sci..

[17] Rossi L., Winands M.H.M., Butenweg C. (2021). Monte Carlo Tree Search as an intelligent search tool in structural design problems. Eng. Comput..

[18] Lehner P., Krejsa M., Pařenica P., Křivý V., Brozovsky J. (2019). Fatigue damage analysis of a riveted steel overhead crane support truss. Int. J. Fatigue.

[19] Nguyen T.-H., Nguyen D.-D. (2020). Reliability Assessment of Steel-Concrete Composite Beams considering Metal Corrosion Effects. Adv. Civ. Eng..

[20] Hohenbichler M., Rackwitz R. (1981). Non-Normal Dependent Vectors in Structural Safety. J. Eng. Mech. Div..

[21] Rosenblatt M. (1952). Remarks on a Multivariate Transformation. Ann. Math. Stat..

[22] Der Kiureghian A., Liu P.-L. (1986). Structural reliability under incomplete probability information. J. Eng. Mech..

[23] Hisada T., Nakagiri S. Role of the Stochastic Finite Element Method in structural safety and reliability. Proceedings of the 5th International Conference on Structural Safety and Reliability.

[24] Liu J.K., Mani A., Belytschko T. (1987). Finite element methods in probabilistic mechanics. Probabilistic Eng. Mech..

[25] Shinozuka M. Basic issues in Stochastic Finite Element analysis. Proceedings of the 5th International Conference on Applications of Statistics and Probability.

[26] Li C.-C., Der Kiureghian A. (1993). Optimal discretization of random fields. J. Eng. Mech. ASCE.

[27] Matthies H.G., Brenner C.E., Bucher C.G., Soares C.G. (1997). Uncertainties in probabilistic numerical analysis of structures and solids-Stochastic finite elements. Struct. Saf..

[28] Pellissetti M.F., Schuëller G.I. (2006). On general purpose software in structural reliability—An overview. Struct. Saf..

[29] Reh S., Beley J.-D., Mukherjee S., Khor E.H. (2006). Probabilistic finite element analysis using ANSYS. Struct. Saf..

[30] Der Kiureghian A., Haukaas T., Fujimura K. (2006). Structural reliability software at the University of California, Berkeley. Struct. Saf..

[31] Schuëller G., Pradlwarter H. (2006). Computational stochastic structural analysis (COSSAN)—A software tool. Struct. Saf..

[32] Thacker B.H., Riha D.S., Fitch S.K.H., Huyse L.J., Pleming J.B. (2006). Probabilistic engineering analysis using the NESSUS software. Struct. Saf..

[33] Gollwitzer S., Kirchgäßner B., Fischer R., Rackwitz R. (2006). PERMAS-RA/STRUREL system of programs for probabilistic reliability analysis. Struct. Saf..

[34] Lemaire M., Pendola M. (2006). Phimeca-soft. Struct. Saf..

[35] Tvedt L. (2006). Proban-probabilistic analysis. Struct. Saf..

[36] Wu Y.-T., Shin Y., Sues R.H., Cesare M.A. (2006). Probabilistic function evaluation system (ProFES) for reliability-based design. Struct. Saf..

[37] Lin H.-Z., Khalessi M. (2006). General outlook of UNIPASS™ V5.0: A general-purpose probabilistic software system. Struct. Saf..

[38] Lee J., Ang A. (1995). Finite element fracture reliability of stochastic structures. Struct. Eng. Mech..

[39] Engelstad S.P., Reddy J.N. (1993). Probabilistic nonlinear finite element analysis of composite structures. AIAA J..

[40] Mahadevan S., Mehta S. (1993). Dynamic reliability of large frames. Comput. Struct..

[41] Zabojszcza P., Radoń U. (2019). The Impact of Node Location Imperfections on the Reliability of Single-Layer Steel Domes. Appl. Sci..

[42] Zabojszcza P., Radoń U. (2020). Stability analysis of the single-layer dome in probabilistic description by the Monte Carlo method. J. Theor. Appl. Mech..

[43] Mochocki W., Radoń U. (2019). Analysis of Basic Failure Scenarios of a Truss Tower in a Probabilistic Approach. Appl. Sci..

[44] Kubicka K., Obara P., Radoń U., Szaniec W. (2019). Assessment of steel truss fire safety in terms of the system reliability analysis. Arch. Civ. Mech. Eng..

[45] Stocki R. (2010). Analiza Niezawodności i Optymalizacja Odpornościowa Złożonych Konstrukcji i Procesów Technologicznych.

[46] Stocki R., Kolanek K., Knabel J., Tauzowski P. (2009). FE based structural reliability analysis using STAND environment. Comput. Assist. Mech. Eng. Sci..

[47] Computer System Numpress. http://numpress.ippt.pan.pl/.

[48] Pettermann H.E., Huber C.O., Luxner M.H., Nogales S., Böhm H.J. (2010). An Incremental Mori-Tanaka Homogenization Scheme for Finite Strain Thermoelastoplasticity of MMCs. Materials.

[49] Berardi V.P., Mancusi G. (2012). Time-Dependent Behavior of Reinforced Polymer Concrete Columns under Eccentric Axial Loading. Materials.

[50] Piotrowski R., Siedlecka M. (2020). Point Protection of Primary Beams of Steel Grillages Against Lateral Torsional Buckling. Adv. Sci. Technol. Res. J..

[51] Xie X., Li X., Shen Y. (2014). Static and Dynamic Characteristics of a Long-Span Cable-Stayed Bridge with CFRP Cables. Materials.

[52] Kubicka K., Pawlak U., Radoń U. (2019). Influence of the Thermal Insulation Type and Thickness on the Structure Mechanical Response Under Fire Conditions. Appl. Sci..

[53] Shi J., Shen J., Yu X., Liu J., Zhou G., Li P. (2019). Stressing State Analysis of an Integral Abutment Curved Box-Girder Bridge Model. Materials.

[54] Barazzetta G.M., Mossa E., Poggi C., Simoncelli M. (2020). The Airplane Hangars of Pier Luigi Nervi: Digital and Scaled Models. J. Int. Assoc. Shell Spat. Struct..

[55] Gwóźdż M., Machowski A. (2011). Selected Studies and Calculations of Building Structures with Probabilistic Methods.

